# Where Symbolisation Fails: Psychoanalytic Perspectives on cPTSD and Personality

**DOI:** 10.1192/j.eurpsy.2026.11725

**Published:** 2026-07-31

**Authors:** P. McGovern

**Affiliations:** Traumepoliklikken, Modum Bad, Oslo, Norway

## Abstract

**Introduction:**

The boundaries between complex post-traumatic stress disorder (cPTSD) and personality disorders, particularly borderline personality disorder (BPD), remain diagnostically and clinically ambiguous. While ICD-11 has introduced cPTSD as a distinct diagnosis, many patients continue to present with overlapping features, challenging rigid categorical distinctions. Psychoanalytic theory offers tools to understand underlying psychic structures — particularly in relation to symbolisation, internal object relations, and the role of enactments.

**Objectives:**

This presentation explores cPTSD and BPD as occupying a transitional diagnostic area. Through psychoanalytic and trauma-theoretical lenses, it seeks to clarify structural, developmental, and transference-based differences between the two, and examine the implications for treatment planning and therapeutic stance.

**Methods:**

Drawing on clinical case material, diagnostic formulation tools, and psychoanalytic conceptual frameworks (Klein, Winnicott, Bion, Kernberg, Joseph, Fonagy), this paper examines:

- The role and failure of symbolisation
- Conflict-based versus deficit-based psychopathology
- Transference and countertransference dynamics
- Distinguishing trauma-related adaptations from primary personality pathology

Visual models are used to illustrate the diagnostic spectrum, symbolisation capacities, and key features of differing personality organisations.

**Results:**

The investigation supports the view that Kernberg’s concept of borderline personality organisation and Bion’s theory of symbolisation are essential in understanding the different needs of these patient groups. The patient’s capacity to symbolise experience — and its presence or absence in the transference — can serve as a key marker in differentiating cPTSD from borderline-level organisation. Assessing the depth of internal object relations, mentalisation, and the patient’s relation to meaning enables a more dynamic understanding of pathology. A spectrum model integrating conflict & deficit axes, alongside levels of symbolisation, offers a more nuanced and clinically useful formulation than traditional diagnostic categories.

**Conclusions:**

Rather than treating complex PTSD and personality disorders as fixed or mutually exclusive categories, this presentation highlights the value of formulation-based, psychoanalytically informed assessment. By attending to levels of symbolisation and personality organisation in the transference, clinicians can better understand what psychic tasks the patient is struggling with — whether mourning, containment, or the construction of meaning. Kernberg’s structural approach and Bion’s insights into mental processing offer powerful tools for identifying the patient’s core difficulties and therapeutic needs. Ultimately, this approach enables us to move beyond classification, toward a clinical stance that honours complexity, supports psychic integration, and promotes development of symbolic capacity.

**Disclosure of Interest:**

None Declared

